# Stakeholder analysis as a tool for sustainable health and welfare interventions: A case of working donkeys in rural and peri-urban areas of Kenya

**DOI:** 10.1017/awf.2026.10096

**Published:** 2026-09-10

**Authors:** Mary N. Gichure, Josphat Gichure, James Kithuka

**Affiliations:** 1 Department of Animal Sciences, Chuka University, Kenya; 2 Chip Meep Growth Hub Ltd; 3 Department of Public Health Pharmacology and Toxicology, University of Nairobi, Kenya

**Keywords:** Animal health, animal welfare, net mapping, stakeholder analysis, sustainability, working donkeys

## Abstract

Addressing the health and welfare challenges that working donkeys face requires a multidimensional, stakeholder-engaged approach. Stakeholders presumed to play a role in the health and welfare of working donkeys were identified through snowball sampling. Focus group discussions (FGDs) and key informant interviews (KIIs) were used to collect qualitative data via unstructured questionnaires to determine linkages, roles, interests, and influences via pairwise ranking, stakeholders’ centrality analysis, and visualisation using NodeXL Basic. The study revealed that 20 stakeholders were involved in providing health and welfare for working donkeys due to their positions and influence in decision-making. Donkey owners and users (0.84), local Non-Governmental Organisations (NGOs) (0.68), Government Department of Veterinary Services (0.42), academic institutions (0.37), and International Non-Governmental Organisations (NGOs) (0.37) were the key stakeholders based on degree centrality. Interactions among the stakeholders were high, as indicated by the average closeness centrality. The users and the local NGO were the gatekeepers to information based on betweenness centrality. Interactions and relationships among stakeholders have a powerful influence on working donkeys and can serve as a foundation for continued engagement through the sharing of perspectives, experiences, and opinions. The need to mainstream donkey health and welfare among other domestic animals in policy development and resource allocation for service delivery is critical. This holistic approach will not only promote the health and welfare of working donkeys and their owners who depend on them for income generation to support their livelihoods but also ensure the long-term viability of the donkeys as a source of employment.

## Introduction

Working donkeys are used to transport goods such as water, livestock feed, and agricultural produce to markets (Gichure *et al.* 2020b). This provides income indirectly through the savings in domestic transport costs (Valette 2014) and directly through charging for a transportation service (Gichure *et al.* 2020b). Income is also obtained via the sale of donkey by-products, such as manure (Ndambi *et al.* 2019) or skin (Wang *et al.* 2014). They also reduce the work burden on women and protect the livelihoods and food security of female-headed households in low- and middle-income countries (LMICs), thereby contributing to household incomes and national economies (Mwasame 2020).

Donkeys continue to be faced with existing and emerging health and welfare challenges, such as being stolen for slaughter, disease incidences, road accidents, and malicious cutting as a result of conflicts (Gichure *et al.* 2020b). They are exposed to poor husbandry and management, as well as injurious working implements such as improper harnesses and cart designs (Brooke 2013). They are also mistreated through whipping, overworking, overloading, poor feeding, and poor handling practices, including the use of tether ropes (Gichure *et al.* 2020b). Donkeys suffer from diseases such as trypanosomiasis (Oduori *et al.* 2015), parasitic infections (Mulwa *et al.* 2020), rabies (Câmara *et al.* 2020), African horse sickness (Gichure *et al.* 2020a), and toxoplasmosis (Obonyo *et al.* 2022), among others. These challenges impact donkeys’ health and welfare as well as their work output, thereby reducing productivity (Gichure *et al.* 2020a; Bonsi *et al.* 2023). Donkey welfare in rural and peri-urban Kenya is generally poor. The donkeys found in urban and peri-urban areas appear to face greater health and welfare challenges than those in rural areas (Kamonzo 2018), with problems stemming from a lack of knowledge, economic constraints, and a low perceived value of the animal. Animal welfare remains an important societal concern (Balzani & Hanlon 2020).

The sustainability of donkey health and welfare interventions in Kenya is threatened by a structural dependency on external donor funding (Haddy *et al.* 2023) and the systemic marginalisation of the species in government resource allocation. This reliance creates a persistent ‘intervention vacuum’ upon donor exit, as donkey owners lack the necessary empowerment and resources to maintain essential husbandry and veterinary standards. The resulting neglect, characterised by delayed disease reporting, financial reluctance toward treatment, and animal abandonment, highlights a breakdown in the care cycle. Consequently, long-term welfare improvements remain elusive without a strategic shift toward state-supported frameworks and community-led self-reliance.

Stakeholder analysis is an approach used to understand a system and its changes by identifying stakeholders and assessing their interest and influence within the system (Hayes *et al.* 2021). Stakeholder engagement has also been used to improve animal welfare across Asia, resulting in improved animal productivity (Sinclair *et al.* 2019). This approach was implemented in Tanzania, to improve animal health surveillance by understanding relevant stakeholders, their interests, and their power. In that study (George *et al.* 2021), strong stakeholder collaboration across the animal health sector reportedly lead to better communication, science, and decision-making, as well as more effective surveillance and response. In Kenya, stakeholder mapping was used to understand animal disease reporting and response in pastoral areas through disease surveillance and timely reporting. In this study, stakeholders’ roles, information flow among them, the methods used for both livestock disease reporting and response, and their strengths and challenges were identified (Sentamu *et al.* 2024).

Insufficient understanding and engagement of key stakeholders involved in the health and welfare of working donkeys make it difficult to define their roles and influence. As a result, valuable support, insights, and feedback may be overlooked, while unanticipated resistance, conflicts, or risks may arise when addressing welfare challenges. Therefore, the potential to improve health and increase productivity in these animals has not been fully realised. Health and welfare problems in donkeys need to be addressed to improve their working efficiency (Bekele et al. 2021), thereby increasing incomes for livelihoods that depend on them (Gichure *et al.* 2020b). This study seeks to determine the contributions of relevant stakeholders to sustainably addressing the current challenges faced by donkeys, as well as to offer an in-depth understanding of these stakeholders, their linkages, and their influence.

## Materials and methods

### Ethical considerations

Research ethical review and approval for this study were obtained from the Chuka University Ethics Committee. Permission was sought from the office of the County Directorate of Veterinary Services, Kirinyaga. Furthermore, informed consent was obtained from all research participants, preceded by an explanation of the study’s objectives and purpose. Plagiarism was checked throughout the study.

### Study area

The study was conducted in Kirinyaga County, Kenya, March and April 2025. The county was purposively selected due to having donkeys working both in rural and peri-urban contexts. Kirinyaga County lies between latitudes 0° 37’S and 0° 45’S and longitudes 37° 14’E and 37° 26’E, at an altitude ranging from 1,158 to 5,199 m above sea level; its highest point being the summit of Mount Kenya. The County has three ecological zones: lowland areas, which lie at 1,158–2,000 m above sea level; midland areas, within 2,000–3,400 m above sea level; and highland areas, at 3,400–5,380 m above sea level. The lowland zone is characterised by gentle rolling plains that cover most of Mwea East and West Sub-Counties. Donkeys occupy these lowland areas. Integrated crop and livestock farming is the main economic activity in the area, which experiences a tropical wet-and-dry or savanna climate, with average daily temperatures ranging from 19 to 33°C across the highlands and lowlands, respectively.

### Study design and data collection

This study adopted a cross-sectional qualitative research design, using focus group discussions (FGDs) and key informant interviews to collect data. A list of 20 potential participants comprising individuals, groups, institutions, or organisations presumed to be knowledgeable or experienced in donkey health and welfare was identified through snowballing (Palinkas *et al.* 2015; Naderifar *et al.* 2017; Kirchherr & Charles 2018).

These participants were engaged in four focus group discussions in the form of stakeholder mapping workshops to identify and map actors involved in working donkeys, their roles, direct and indirect contributions to working donkeys, their interactions and influence in decision-making, and provision of technical support for donkey health and welfare. FGD cross-participant discussion was encouraged through open-ended questions, which led to more in-depth and honest data collection (Sinclair *et al.* 2019). The guiding questions during these workshops were: (i) which stakeholders were involved with the health and welfare of working donkeys in Kirinyaga County? (ii) What roles did they play in the promotion of donkey health and welfare? (iii) How did they interact, and (iv) how did they influence decision-making in relation to health and welfare? Net-mapping was used, in which participants could visualise the interaction and direction in a flow diagram using different colours, linkages, and levels of influence and power (Schiffer & Hauck 2010). Key actors were represented as nodes, while relationships and interactions were represented as lines on the Net-Map flow diagram. The interactions were based on the flow of finances, human resources, information, technical expertise, linkages, training, and potential influence within the working environments. Pairwise ranking was then performed to visualise the relative influence of one actor compared to another. This was done by asking who was more important in influencing an action than the others. This resulted in a two-dimensional matrix/grid with importance and influence or power and interest as its axes (Bryson 2004), which was used to prioritise the actors.

Key informant interviews were carried out to triangulate data collected through FGDs (Caillaud & Flick 2017). Participants identified through snowballing who were unable to participate in the FGDs due to their availability were engaged as KIIs.

### Data analysis

The Net-Map tool provided a flow diagram with nodes and linkages, which revealed a qualitative visual analysis of the networks (Leimkühler *et al.* 2024). Data were entered into a Microsoft Excel® spreadsheet for analysis. It was triangulated and collated by cross-referencing qualitative data from the FGDs and KIIs (Carter *et al.* 2014).

A five-point Likert rating scale was adopted to investigate the level of interest and influence among stakeholders (Jokonya *et al.* 2015). The level of interest was considered to be how much attention the stakeholders were paying to ensuring the optimum health and welfare of the donkeys, and it was assessed at a five-point Likert-type score where 1 = disinterest, 2 = low interest, 3 = moderate interest, 4 = high interest, and 5 = exceeding interest. The level of influence was considered the power stakeholders had over efforts to implement health and welfare provisions for the donkeys. This was also assessed using a five-point Likert-type scale where (1 – not influential, 2 – low influence, 3 – moderate influence, 4 – strong influence, and 5 exceeding influence). This was computed in NodeXL Basic (Smith 2013).

For this study, stakeholder importance was assessed based on their level of influence (Magassouba *et al.* 2019; Hayes *et al.* 2021). The stakeholders were divided into two sections: (1) major stakeholders which included those with a high level of influence; and (2) minor stakeholders, which included those with a low level of influence. Social network analysis (SNA) was used to analyse and visualise the relationships and influences of key stakeholders (Ahmadi *et al.* 2019; Skibski *et al.* 2019). Centrality measures, including degree centrality (undirected, in-degree, and out-degree), closeness centrality, and betweenness centrality (Wu *et al.* 2020) were generated in Gephi version 0.10.1 with the network statistics normalised (Bastian *et al.* 2009). The networks were presented using the Yifan Hu network layout algorithm (Hu 2012).

Degree centrality was used to quantify a node’s relative power based on the number of connections it had and the number of other nodes it could directly communicate with. It served as a measure of stakeholder interconnectedness and was expressed as a value ranging from 0 to 1, where 0 represented the least-connected node, and 1 represented the most highly connected node (Golbeck & Klavans 2015). Closeness centrality was used to assess the average distance of a stakeholder to all other stakeholders in the network, indicating how efficiently information could be disseminated from that node. The measure was normalised and expressed on a scale from 0 to 1, with higher values indicating a more central and strategically important position within the network (Hansen *et al.* 2011). Betweenness centrality was used to quantify the extent to which a stakeholder lay on the shortest paths between other pairs of nodes, indicating how frequently connections passed through that stakeholder. This measure captured the stakeholder’s potential influence over information flow within the network. Betweenness centrality values were normalised to the range 0–1, with higher values indicating greater influence and control over the dissemination of information across the network (Newman 2005; Vignery & Laurier 2020).

## Results and Discussion

### Stakeholders and their roles in donkey health and welfare in Kenya

To enhance animal health and welfare outcomes, twenty key stakeholders were identified across a broad spectrum, including donkey owners and users, government agencies, industry representatives, NGOs, and academic institutions. These participants held diverse roles that dictated their influence over resource allocation, information exchange, and critical decision-making processes regarding working donkeys. Table 1 presents the identified stakeholders involved in working donkeys’ health and welfare and their roles.

**Table 1.** The roles of stakeholders (n = 20) participating in a focus group on the health and welfare of working donkeys in Kenya Table 1. long description.

S. No	Key Actor	Role in donkeys’ health and welfare
1	Donkey owners and users	Cited as the primary decision-makers responsible for the overall welfare of their donkeys Provided for the basic needs of donkeys, including food, water, shelter, and rest. Actively participated in ensuring donkey health and welfare through routine care, monitoring, and seeking veterinary interventions when needed Determined working conditions, including loading limits, riding practices, and daily labour schedules, which directly influenced donkey health, safety, and productivity
2	Local NGO	Provide emergency and specialised treatment for donkeys, Provided guidance and technical advice to stakeholders, including donkey owners, community leaders, artisans, and local authorities on best practices in donkey health and welfare. Facilitated knowledge transfer and capacity building across the donkey welfare network, including training animal health service providers on disease treatment and hoof care, educating donkey owners and users on basic first aid, home-based care, husbandry requirements, and promoting best practices in disease prevention, humane handling, safe loading, and use of working implements. Acted as resource hubs by sharing evidence-based information, training materials, and updates on welfare standards to improve decision-making across the stakeholder network. Supported coordination among stakeholders to align welfare practices and promote a consistent, community-wide approach to donkey health and safety. Trained artisans on the design and production of appropriate donkey carts and harnesses using evidence-based technologies.
3	Government Veterinary Department	Collaborate with local NGOs to provide emergency and specialised disease treatment for donkeys Provided training to donkey owners on donkey health and management. Official reporting channel for notifiable animal diseases
4	Education institutions	Provided awareness and training to pupils and students on equine welfare through donkey care clubs, animal welfare clubs, and integration into formal curricula
5	International NGO	Funded local NGO activities to support donkey health and welfare initiatives. Advocated for the inclusion of donkey welfare in policy agendas and media platforms.
6	LAHPs (Local animal health providers) and farriers - Private	provided treatment for sick donkeys and performed hoof trimming. Supplied donkey owners with health care products, including medicines
7	Donkey Owners CBO (community-based organisations)	Served as entry points for NGOs to implement group-based interventions. Developed welfare-friendly by-laws guiding donkey management and working conditions. Represented a unified voice to advocate for donkey needs to other stakeholders.
8	National government	Coordinate all County governments in the provision of animal health and welfare activities Issue licences of operation to local animal health providers and NGOs
9	County Government	Formulate by-laws that guide the use of donkeys, especially in the town areas such as markets, roadside waiting bays
10	Government Livestock Production Department	Through extension officers, provided training and advisory services to donkey owners and users on appropriate husbandry practices, including feeding, watering, housing, rest, and workload management.
11	Customers - Service Beneficiaries	Drove demand for healthy donkeys by preferring services from visibly healthy, fit, and well-managed donkeys, indirectly motivating owners to maintain donkey health and welfare
12	Chiefs - Village Administrators	Managed community-level conflict resolution related to donkeys, such as straying and property damage. Managed community-level conflict resolution related to donkeys, such as straying and property damage.
13	Police Department	Enforced animal welfare legislation, including the Prevention of Cruelty to Animals Act (CAP 360). Sensitised donkey owners and users on road safety through collaborative training sessions coordinated by NGOs Responded to reports of donkey abuse, overloading, neglect, or cruelty in collaboration with veterinary officers and local administrators. Supported investigations and prosecution of donkey welfare offenders through the legal system
14	Law Courts - Magistrate	Make judgments and punish donkey welfare offenders under the Prevention of Cruelty to Animals Act (CAP 360), ensuring accountability for abuse, neglect, or overloading. Oversee prosecutions in collaboration with police and veterinary officers, reinforcing compliance with welfare standards and promoting a safer working environment for donkeys
15	Artisans	Produced, repaired, and maintained donkey carts and harnesses, reducing the risk of injuries and improving the safety and comfort of working donkeys.
16	Merchants	Selected healthy donkeys at the market to resell to owners, ensuring the animals were immediately fit for work and could perform tasks effectively.
17	Social Services Department	Trained owners and users on the risks of drug and substance abuse. Addressed social factors that could negatively affect donkey welfare, promoting responsible care practices.
18	Media	Raised awareness about donkey health and welfare, influenced community norms, and provided a platform for advocacy
19	Public Health Department	Formulated bylaws to guide donkey use in public spaces, such as markets and water collection points, creating a safer and more structured working environment that reduces community conflicts
20	Community	Acted as whistleblowers by reporting cases of neglected or mistreated donkeys, enabling early interventions.

**Figure 1.**
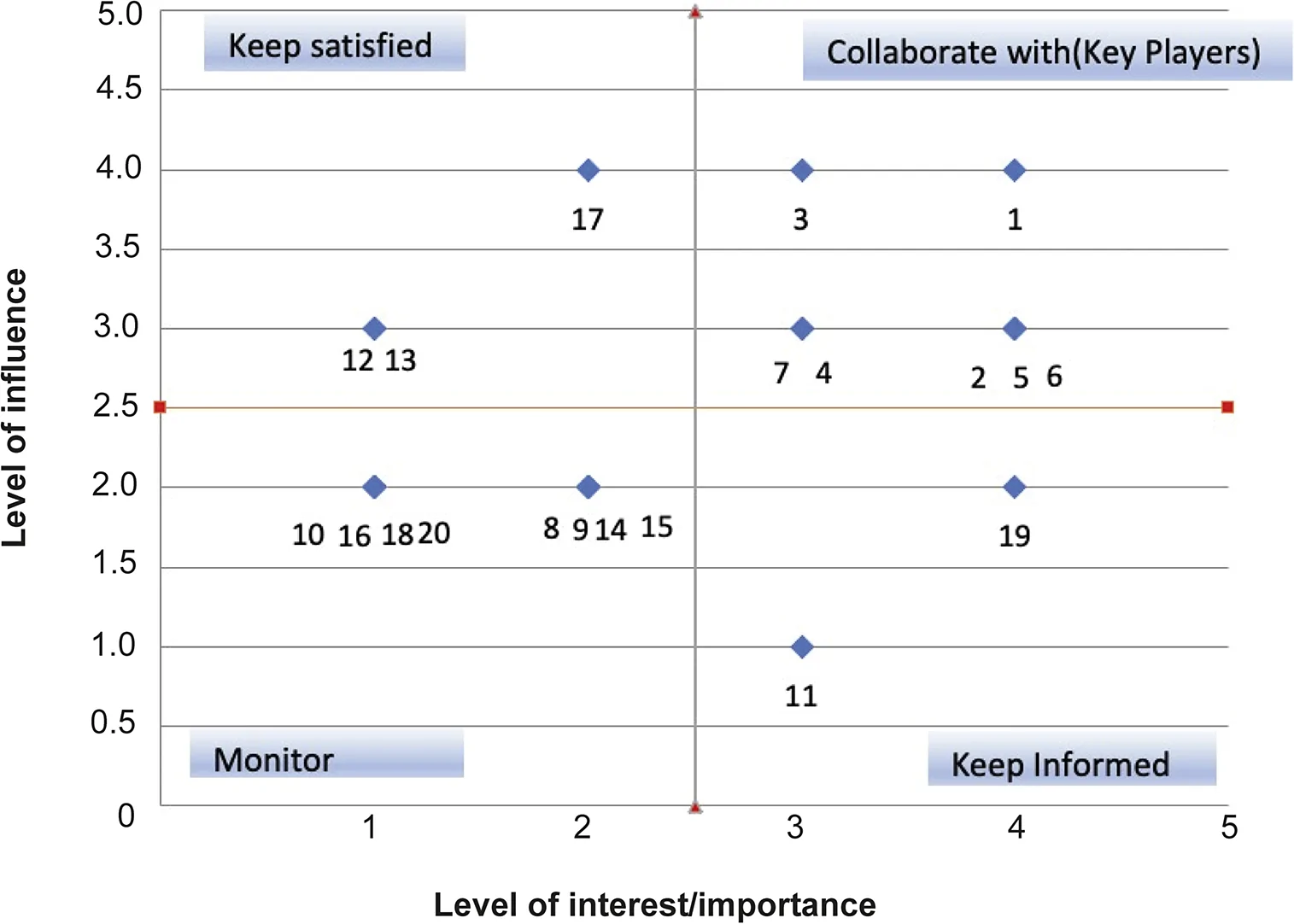
Stakeholder power-influence mapping using participatory methods in the working donkey sector (n = 20), Kenya. 1-Donkey Owner and User; 2-Local NGO; 3-Govt Vet Department; 4-Academic institutions; 5-International NGO; 6-Donkey Owners CBO; 7-LAHPs and farriers - Private; 8-Govt Livestock Production Department; 9-Village chiefs (local administrators); 10-Police Department; 11-Community; 12- Local/County Government; 13-Central/National Government; 14-Merchants; 15-Media; 16-Public Health department; 17-Customers (Service Beneficiaries); 18-Law Courts (Magistrate); 19-Artisans; 20-Social Services Department.

Stakeholders were mapped using a five-point Likert scale to assess their levels of interest and influence regarding donkey health and welfare and were categorised into four distinct groups. The results of this mapping are presented in Figure 1. Stakeholders were mapped into four categories: (i) low-interest, low-influence stakeholders, who required monitoring; (ii) low-interest, high-influence stakeholders, who needed to be kept informed; (iii) high-interest, low-influence stakeholders, who should be kept satisfied; and (iv) high-interest, high-influence stakeholders, who were key actors collaborating to ensure optimal health and welfare for working donkeys. Considering that influence was deemed more important in this study (Hayes *et al.* 2021), stakeholders were further grouped into two broader classifications: (1) major stakeholders, with a high level of influence; and (2) minor stakeholders, with a low level of influence.

The key actors identified in this study comprised donkey owners and users, local non-governmental organisations, government veterinary departments, educational institutions, international non-governmental organisations, community-based organisations, and local animal health providers and farriers. Similarly, other studies identified animal owners, veterinarians, industry associations, research and development bodies, funders, industry consultants, agribusinesses, and consumers as key stakeholders (Fernandes *et al.* 2019).

The recognition of animal owners as the key actors in implementing animal welfare cannot be overstated (Balzani & Hanlon 2020).

Donkey owners and users were the primary decision-makers responsible for providing for their donkeys’ basic needs and seeking veterinary care when required. Their interests are largely economic, as they rely on donkeys for income, transport, and labour. Many owners are resource-poor and may lack the knowledge or financial means for proper veterinary care, nutrition, or equipment. Interventions must therefore consider their economic realities and focus on sustainable, low-cost solutions. Understanding how owners perceive and value donkey health and welfare is essential for designing effective programmes and policies that maintain healthy, productive animals (Balzani & Hanlon 2020). Their attitudes and practices have a direct impact on donkey welfare. In particular, their knowledge, skills, and abilities are key factors that determine how effectively behaviour change can be implemented among animal owners (Peden *et al.* 2018). This highlights the importance of continuously generating and transferring knowledge to donkey owners. Knowledge can be shared by experts, such as animal health professionals, providing a trusted source of information, or through peer-to-peer learning, which offers authenticity and can help shape social norms among owners. Such knowledge is essential for enabling donkey owners to make informed decisions that improve the health and welfare of their animals.

Donkey owners’ community-based organisations (CBOs) were the primary form of grouping in this study. The CBOs offered several benefits. First, they promoted peer-to-peer accountability and motivation to improve donkey health and welfare; indeed, Sinclair *et al.* (2019) noted that farmers are often more motivated by their peers than by other stakeholders. Second, CBOs served as an entry point for NGOs to deliver knowledge-change training, as it was easier to train donkey owners who were organised into groups. Third, CBOs united donkey owners and enabled them to lobby collectively for improved working conditions for their donkeys. This finding is consistent with Omollo *et al.* (2019), who reported that working in groups allows producers to more easily access technical support, information, linkages, and interaction with other stakeholders along the value chain.

Academic institutions were identified as key actors in this study. Their primary role was to generate evidence-based knowledge and practical approaches to improving donkey health and welfare. These institutions facilitated the formal exchange of technical training, guidelines, information, research evidence, routine data, and field-based experiences or case studies (Schneider *et al.* 2022). That information would then be integrated into the teaching curriculum to equip students with the knowledge and skills needed to address these issues, ensuring they are adequately prepared before entering professional practice. This approach has been successfully implemented in the UK as part of its animal health strategy initiatives (Muldoon & Williams 2021). The information would ultimately reach the donkeys through animal health providers, whose capacity to treat them and provide disease-prevention advice would have been strengthened.

The stakeholders who contributed financial resources comprised the international and local NGOs, were considered key actors. Their primary interest was improving animal welfare through education, veterinary services, advocacy, and research. This category appeared to include all other stakeholders, as it provided financial support during project implementation. This observation was also noted by Sentamu *et al.* (2024) in a similar Kenyan context, where they identified NGOs’ roles in collaboration and in supporting County Governments with resources to manage animal disease outbreaks. The NGOs also offered training to local animal health providers on donkey health and welfare and provided linkages to suppliers of equine-specific equipment, such as hoof-care equipment, at subsidised cost. They also connected these local animal health providers with donkey owners to provide disease healthcare. This perceived influence, however, needed to be assessed in light of sustainability when NGOs exit an operational area. The financial support of NGOs cannot be wished away since an under-resourced stakeholder network has been shown to limit the power to execute its objectives (Fernandes *et al.* 2019). As such, their real influence should be evaluated to ensure they do not gain too much influence over decisions regarding animal health and welfare, which could compromise the overall sustainability of health and welfare provision (Muluh *et al.* 2019; Haddy *et al.* 2023). The adoption of public-private partnerships between the government and the NGOs could provide an opportunity for collaboration in donkey health and welfare (N’Guessan *et al.* 2022).

Local animal health practitioners were identified as key actors in this study. These were the frontline service providers. Their interest was in the health of donkeys, focusing on disease prevention (through vaccination), the treatment of sick donkeys, and routine management procedures such as hoof care (for farriers), as well as advising on feeding, watering, and donkey welfare. Their competence was enhanced by veterinary staff from international and local NGOs, who continually built their capacity to handle donkey cases. These local animal health practitioners utilised a working system of referral and case reporting with the government directorate of Veterinary Services (Kahariri *et al.* 2024), following the privatisation of animal healthcare provision in Kenya (Chema & Gathuma 2004).

Both national and county governments were identified as key actors in this study, playing a dual role in donkey health and welfare. First, the national government, through the Directorate of Veterinary Services, was responsible for economic development and livestock management, including animal health activities. Second, the County government of Kirinyaga, which provided oversight through the development, regulation, and enforcement of livestock health and welfare policies at the local level. These roles were crucial within collaborative stakeholder networks, as governments have the authority to coordinate stakeholders, mobilise resources, and shape policy. Their engagement also enabled the integration of donkey welfare into broader livestock and agricultural strategies, strengthened legislation at both national and county levels, including through county-specific bylaws, and increased public confidence in donkey welfare initiatives. For example, public-private partnerships between governments and NGOs facilitated animal welfare projects, culminating in policy developments such as the Animal Welfare and Protection Bill, 2019. At the time of preparing this manuscript, the bill remained pending, awaiting cabinet and parliamentary legislative enactment. While involving government stakeholders is valuable, implementation can be slowed by lengthy deliberation processes, shifts in political leadership priorities, detailed reporting requirements, and limited flexibility when other scheduled activities take precedence, as has been previously observed (Head 2005). Therefore, it is essential to keep these stakeholders regularly informed to ensure that policies and laws remain relevant and adaptable, supporting long-term gains despite changes in political leadership.

The findings in this study revealed that customers, also referred to as service beneficiaries, had a strong influence on the health and welfare of donkeys. It was interesting to note that customers (service beneficiaries) preferred to have only healthy donkeys carrying their goods; therefore, their demand for transport services would affect the income earned for the donkey owner. As such, the improvement of animal welfare was essential to meet customer demands and expectations (Hansson & Lagerkvist 2015; Sinclair *et al.* 2019).

Focusing solely on animal welfare is unlikely to yield positive outcomes if the underlying issues relate to the well-being and socioeconomic status of the animal-owning community. A holistic approach that links animal and human welfare has been advocated, embodied in the One Welfare concept, which emphasises addressing both simultaneously (Pinillos *et al.* 2016). As Mahatma Gandhi famously stated, “*The greatness of a nation and its moral progress can be judged by the way its animals are treated*,” highlighting that a society’s treatment of animals reflects its moral values and social understanding. In Kenya, the Social Services Department provides support to donkey owners and users on issues such as substance abuse, which is prevalent in some communities. Certain donkey welfare challenges, such as excessive whipping, may stem from the owners’ social or personal issues, including mental health challenges, learned behaviours, or substance abuse. Studies have shown that human health and social determinants can directly influence animal welfare, particularly among companion animals. (McDowall *et al.* 2023) and production animals (Sinclair *et al.* 2019). Studies relating this association among large animals, especially working equines are, however, scarce.

Stakeholders who had the least direct contact with donkey owners and users regarding health and welfare included village chiefs and administrators, the police, law courts and magistrates, the public health department, merchants, and the media. Although these stakeholders were categorised as having low influence and low interest, they nonetheless helped create a safer and more supportive environment for donkeys and their users, both on the roads and within the community. Animal welfare in Kenya is legally addressed under the Prevention of Cruelty to Animals Act CAP 360 (1962). Police officers and magistrates in the courts are mandated to enforce animal-related laws and bylaws (Rault *et al.* 2022). Merchants and artisans were involved in the production, crafting, and sale of working implements such as donkey carts and harnesses. However, the suitability of these implements for donkeys working across diverse terrains, such as murram and tarmac roads, as well as hilly areas, was rarely documented. Consequently, no standard cart design was recommended for working donkeys, resulting in a wide variety of carts and harnesses across different areas of the county. These variations in equipment were potential contributors to wounds and other injuries, thereby negatively affecting donkey health and welfare, as previously reported by Rayner *et al.* (2020). Although merchants and artisans were classified as minor stakeholders, their contributions to donkey health and welfare, particularly through the design and provision of suitable working implements, remain significant. The media, on the other hand, can play an important role by raising awareness, influencing policy, and promoting a more humane society, although this role was not emphasised in the present study.

During focus group discussions and key informant interviews in this study, minor stakeholders did not perceive themselves as having a direct impact on donkey health and welfare. However, their perspectives may evolve in response to the dynamic working environment and a more holistic understanding of welfare. Therefore, it is important to maintain ongoing engagement with these stakeholders through regular dialogue and monitoring. It should also be noted that stakeholder roles and influence can change over time, as new actors emerge and others become less relevant, highlighting the need for continuous stakeholder analysis.

The computed centrality measures are presented in Table 2.

**Table 2.** Centrality measures of a stakeholder network (n = 20) within the Health and Welfare Sector of Working Donkeys in Kenya Table 2. long description.

Id	Label	Closeness	Betweenness	In Degree	Out Degree	Degree Undirected
1	DOU	0.86	0.45	0.79	0.84	0.84
2	LNGO	0.76	0.23	0.84	0.68	0.68
3	GVET	0.63	0.03	0.42	0.42	0.42
4	EDU	0.61	0.01	0.42	0.37	0.37
5	INGO	0.54	0.02	0.42	0.37	0.37
6	CBO	0.56	0	0.21	0.21	0.21
7	LAHP	0.56	0	0.26	0.26	0.26
8	GLPD	0.59	0	0.32	0.32	0.32
9	VCA	0.57	0.01	0.21	0.26	0.21
10	PD	0.57	0.02	0.21	0.26	0.26
11	COMM	0.54	0.01	0.21	0.21	0.21
12	CGOV	0.56	0.02	0.21	0.21	0.21
13	NGOV	0.57	0.05	0.47	0.42	0.42
14	MECH	0.54	0	0.11	0.21	0.16
15	MEDI	0.46	0	0.16	0.16	0.16
16	PHD	0.5	0	0.11	0.11	0.11
17	CSB	0.47	0	0.05	0.05	0.05
18	LAWC	0.47	0	0.16	0.16	0.16
19	ART	0.51	0	0.11	0.11	0.11
20	SSD	0.47	0	0.05	0.05	0.05

Key/ Legend: DOU-Donkey Owner and User; LNGO-Local NGO; GVET-Govt Vet Department; EDU-Education institutions; INGO-International NGO; CBO-Donkey Owners CBO; LAHPs- Local Animal Health Practitioners- Private; GLPD-Government Livestock Production Department; VCA-Village chiefs - Administrators; PD-Police Department; COMM-Community; NGOV National government; CGOV County Government; MECH-Merchants; MEDI-Media; PHD-Public Health department; CSB-Customers - Service Beneficiaries; LAWC-Law Courts (Magistrate); ART-Artisans; SSD-Social services Department.

### Participatory mapping of stakeholder networks and linkages within the Kenyan donkey health and welfare sector

**Figure 2.**
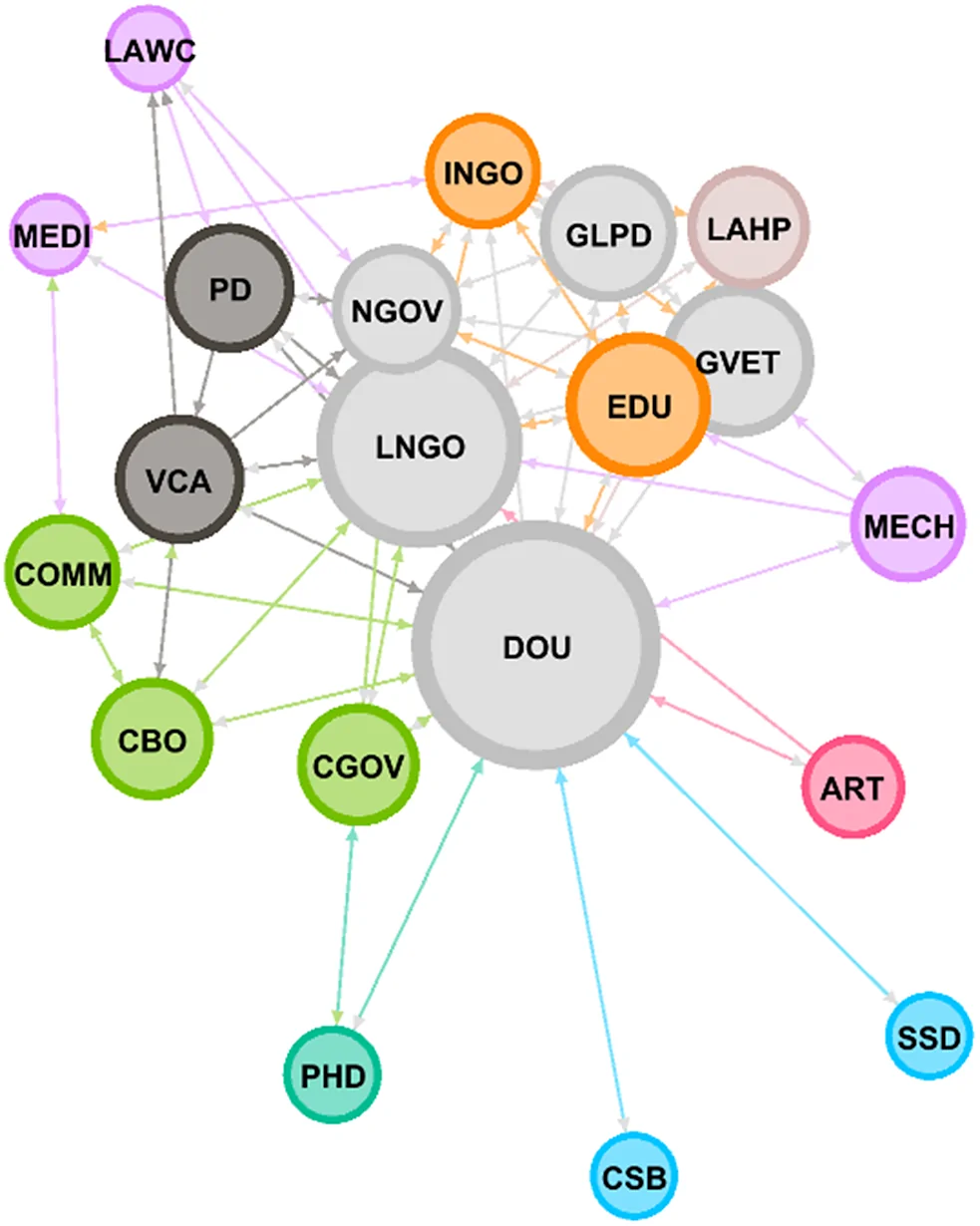
NetMap visualisation of closeness centrality within the stakeholder network (n = 20) for the health and welfare of working donkeys in Kenya.

**Figure 3.**
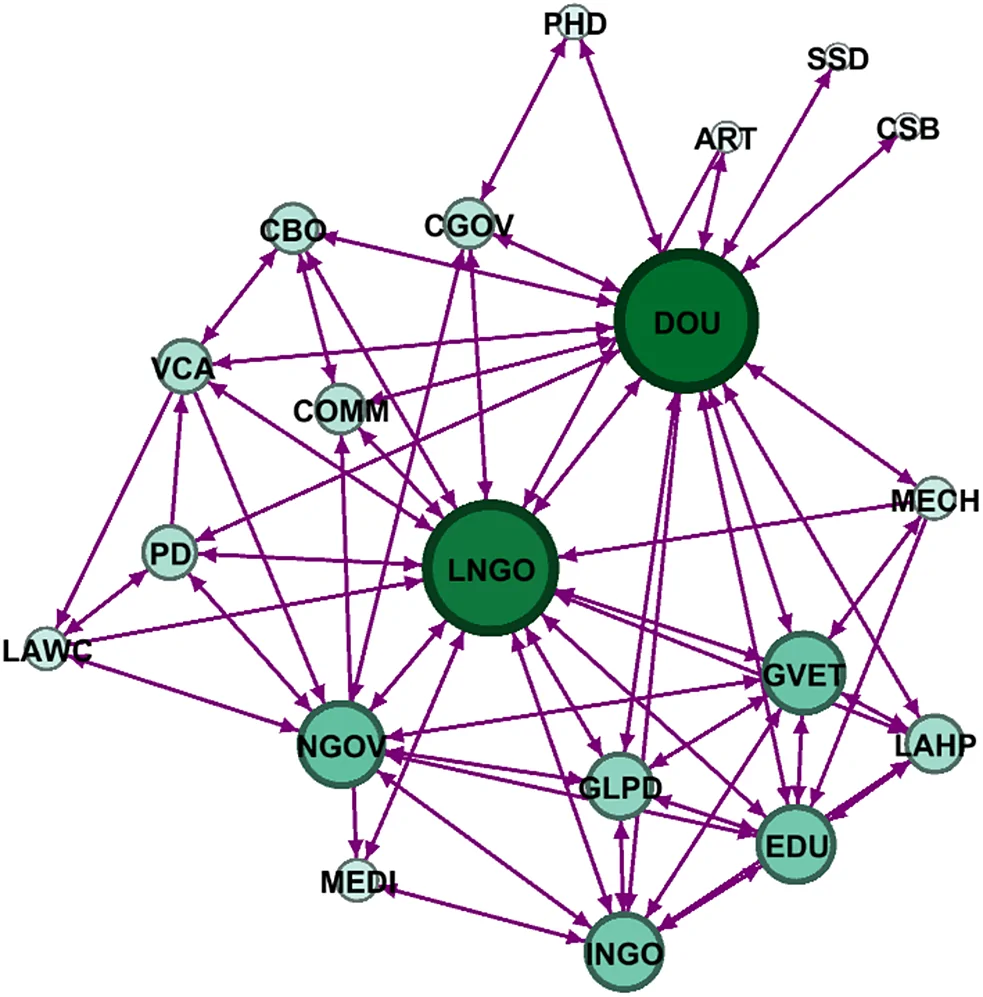
NetMap visualisation of degree centrality within the stakeholder network (n = 20) for health and welfare of working donkeys in Kenya.

**Figure 4.**
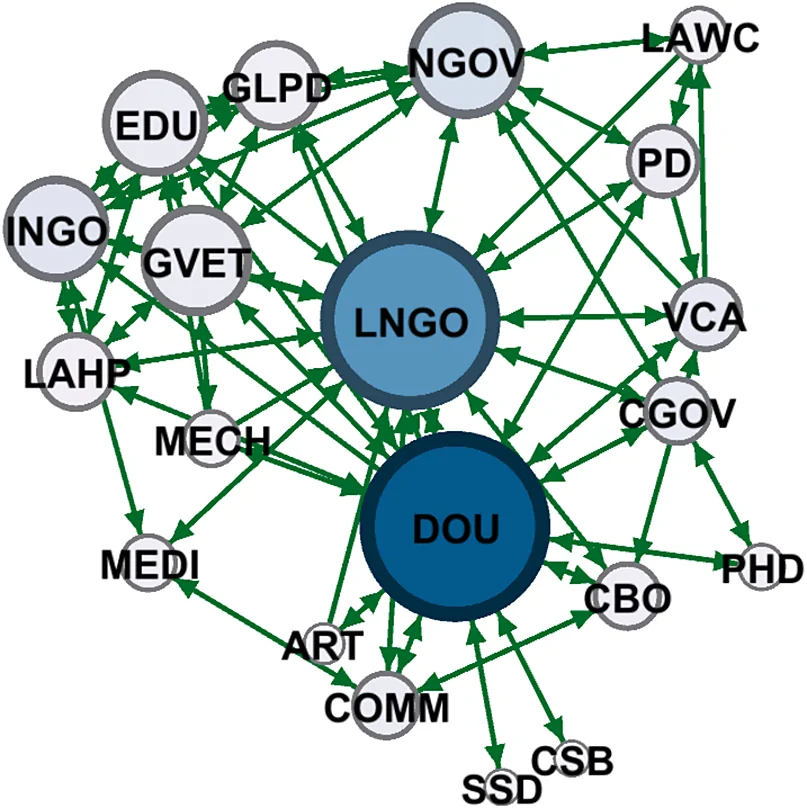
NetMap visualisation of betweenness centrality within the stakeholder network (n = 20) for health and welfare of working donkeys in Kenya.

Stakeholder flow diagrams showing stakeholders (nodes) and their interactions (linkages) flow of information, financial resources, training, and technical support are presented in Figures 2, 3 and 4, according to their centrality measures (refer to the abbreviations presented in Figure 1). The size of the nodes was proportional to the centrality values they represented.

The closeness centrality for the working donkeys’ stakeholder network is presented in Figure 2.

Within the donkey health and welfare sector, closeness centrality measures identified donkey owners and users as the primary influencers (0.86), followed by local NGOs (0.76), government veterinary services (0.63), and educational institutions (0.61). With an aggregate network mean of 0.56, these values indicate a high degree of connectivity and cohesion among stakeholders (Nyokabi *et al.* 2023). Such results suggest that the network structure supports efficient interactions, as high closeness values reflect shorter paths for communication and technical support between nodes (Uddin *et al.* 2023).

For this network, degree centrality was directed. The donkey owners and users (0.79) and the local NGOs (0.84) had the highest in-degree centrality. These two stakeholders also had the highest out-degree centralities, at 0.84 and 0.68, respectively. The in-degree centrality indicates a particular actor’s tendency to receive inputs from other actors in the network, thus measuring the actor’s receptivity or popularity (Puri *et al.* 2017). As such, these stakeholders were actively engaged in improving donkey health and welfare.

The degree centrality (undirected) was also computed and visualised as presented in Figure 3. Donkey owners and users had the highest score, at 0.84, followed by the local NGO at 0.68 and the government veterinary services department at 0.42. This indicated that they had the greatest ability to perform their various tasks in ensuring optimal health and welfare for the donkeys, including meeting basic husbandry requirements, providing health services to the donkeys, and training the owners and users on various aspects of managing their donkeys. These findings corroborate those of Sentamu *et al.* (2024), who also showed that animal owners were the most valuable stakeholders in ensuring optimal health for their animals. Therefore, any intervention towards improving donkey health and welfare should involve the donkey owners.

Further, betweenness centrality was also computed and visualised in Figure 4. Interestingly, the donkey owners and users (0.45) and the local NGO (0.23) had the highest betweenness centrality values. These two were the gatekeepers and highly influential stakeholders in the network due to their central position in connecting other stakeholders.

### Animal welfare implications

While donkey welfare in Kenya is compromised by insufficient knowledge, economic constraints, and the species’ low socio-economic status, stakeholder mapping offers a sophisticated solution. Rather than addressing these issues superficially, this mapping approach facilitates a deeper analysis of the intricate relationships, underlying motivations, and power dynamics that dictate animal health outcomes. By systematically identifying and analysing stakeholders, efforts to improve donkey health and welfare could prove more sustainable, targeted, effective, relevant, ethical, culturally appropriate, and ultimately successful. The failure to address the diverse needs and interests of these stakeholders could lead to stagnation of efforts to improve animal welfare.

While identifying stakeholders is a critical first step, translating engagement into tangible improvements in donkey welfare requires deliberate coordination, clearly defined roles, and actionable mechanisms. Major stakeholders, including government agencies, NGOs, and veterinary professionals, alongside minor stakeholders such as merchants, artisans, and community leaders, must collaborate to implement disease prevention measures, promote safe working practices, facilitate knowledge transfer, and enforce policies. Engagement should occur through both formal channels, such as training sessions, workshops, and multi-stakeholder committees, and informal peer-to-peer networks, reinforced by continuous feedback, monitoring, and adaptive management. Multi-stakeholder platforms facilitate joint decision-making, capacity building, and the promotion of social norms that support welfare, while aligning interventions with owners’ economic realities and harmonising policies across sectors, all of which are supported by legal frameworks, such as CAP 360. Measurable outcomes, including reduced disease incidence, lower injury rates, prompt veterinary interventions, and safer working environments, can thus be directly linked to stakeholder actions. By fostering trust, collaboration, and a culture of continuous learning, stakeholder engagement moves beyond identification to ensure evidence-based, practical, and economically viable solutions, ultimately achieving sustainable improvements in the health and welfare of working donkeys.

## Conclusion

This study highlighted the challenges faced by working donkeys while also providing a sustainability orientation to address their health and welfare, thereby offering both a theoretical and empirical contribution to the topic through stakeholder engagement.

The involvement of key stakeholders presents a sustainable approach to addressing donkey health and welfare challenges, despite its apparent complexity. This complexity calls for continual engagement with stakeholders to regularly review challenges, clarify their roles, assess strategies, and track the progress of activities implemented to improve the health and welfare of donkeys. The success of sustainable health and welfare provision for working donkeys depended on the participation of donkey owners and users, as well as on coordination among local NGOs. Interestingly, this study revealed existing interactions and relationships among stakeholders, as evidenced by high closeness centrality measures. This could have a powerful influence on working donkeys and serve as a foundation for future engagements. Continued engagement with major stakeholders by incorporating their perspectives, experiences, and opinions helps them feel involved, appreciate their contributions, and take on their roles to fully address the challenges in the dynamic working environment.

A critical question remains regarding the primary driver of equine welfare in Kenya: who bears the ultimate responsibility for ensuring optimum health and welfare standards? At present, coordination efforts are predominantly led by NGOs, highlighting a potential gap in public sector involvement and long-term institutional leadership. To enhance the sustainability of donkey health and welfare, this study recommends strengthening public-private partnerships (PPPs) to foster collaboration between government bodies and NGOs. By activating broader stakeholder networks, the current fiscal over-reliance on the NGO sector can be mitigated, improving long-term institutional stability. Furthermore, governments must mainstream donkey welfare into national policy and resource allocation frameworks alongside other livestock. Such a holistic strategy not only safeguards the health of these animals but also secures the livelihoods of owners who depend on donkeys for income generation and employment.

## Long descriptions

### Table 1. Long description

The table consists of three columns: S. No, Key Actor, and Role in donkeys’ health and welfare.

1. Donkey owners and users: Primary decision-makers providing basic needs, routine care, and determining working conditions.

2. Local N G O: Provides emergency treatment, technical advice, capacity building, and trains artisans on cart design.

3. Government Veterinary Department: Collaborates with N G Os for treatment, trains owners, and acts as an official reporting channel for diseases.

4. Education institutions: Provides awareness to students through donkey care clubs and curricula.

5. International N G O: Funds local N G O activities and advocates for policy inclusion.

6. L A H P s (Local animal health providers) and farriers - Private: Provides medical treatment, hoof trimming, and health care products.

7. Donkey Owners C B O (community-based organisations): Acts as entry points for interventions, develops by-laws, and represents a unified voice.

8. National government: Coordinates County governments and issues operating licenses.

9. County Government: Formulates by-laws for donkey use in town areas and markets.

10. Government Livestock Production Department: Provides training on husbandry practices through extension officers.

11. Customers - Service Beneficiaries: Drives demand for healthy donkeys, motivating owners to maintain welfare.

12. Chiefs - Village Administrators: Manages community-level conflict resolution regarding straying or damage.

13. Police Department: Enforces welfare legislation (C A P 360), sensitizes on road safety, and responds to abuse reports.

14. Law Courts - Magistrate: Makes judgments and punishes offenders under C A P 360.

15. Artisans: Produces and maintains carts and harnesses to reduce injury risks.

16. Merchants: Selects healthy donkeys at markets for resale.

17. Social Services Department: Trains owners on risks of drug abuse and addresses social factors affecting welfare.

18. Media: Raises awareness and provides a platform for advocacy.

19. Public Health Department: Formulates by-laws for public spaces like water collection points.

20. Community: Acts as whistleblowers for cases of neglect or mistreatment.

Navigate back to Table 1..

### Table 2. Long description

The table contains seven columns: Id, Label, Closeness, Betweenness, In Degree, Out Degree, and Degree Undirected.

Key data points include:

* D O U (Donkey Owner and User) has the highest scores across most metrics: Closeness 0.86, Betweenness 0.45, In Degree 0.79, and Out Degree 0.84.

* L N G O (Local N G O) follows with Closeness 0.76, Betweenness 0.23, and the highest In Degree at 0.84.

* N G O V (National Government) shows moderate influence with Closeness 0.57 and In Degree 0.47.

* Several stakeholders, including C B O, L A H P, G L P D, M E C H, M E D I, P H D, C S B, L A W C, A R T, and S S D, have a Betweenness score of 0.

* The lowest overall engagement is seen in C S B (Customers - Service Beneficiaries) and S S D (Social Services Department), both with In Degree and Out Degree scores of 0.05.

* Closeness values range from a high of 0.86 (D O U) to a low of 0.46 (M E D I).

Navigate back to Table 2..
